# Immune checkpoint inhibitor therapy for primary neuroendocrine carcinoma of the gallbladder: A case report and literature review

**DOI:** 10.1097/MD.0000000000040178

**Published:** 2024-10-25

**Authors:** Chao Li, Pan Lv, Liu Yanyan, Maohui Yin, Hao Li

**Affiliations:** a Hepatobiliary and Pancreatic Medicine Center, Weifang Peoples Hospital, Weifang, Shandong, P.R. China.

**Keywords:** gallbladder neuroendocrine carcinoma, GB-NEC, immune checkpoint inhibitors, immunocytochemistry, neuroendocrine tumors

## Abstract

**Rationale:**

Gallbladder neuroendocrine carcinoma (GNEC) is indeed a relatively rare malignant tumor of the gallbladder with neuroendocrine differentiation and the ability to produce and secrete a number of neurotransmitters and hormones, characteristics that make its clinical presentation and biological behavior likely to be different from those of other types of gallbladder cancer. Current treatment mostly relies on surgery and adjuvant chemotherapy and radiotherapy.

**Patient concerns:**

We report a 53-year-old middle-aged male patient who underwent radical surgery for gallbladder malignancy after a diagnosis of neuroendocrine carcinoma of the gallbladder.

**Diagnoses:**

Diagnosis of neuroendocrine carcinoma of the gallbladder based on the return of pathologic findings.

**Intervention:**

After local progression of postoperative chemotherapy with the first-line regimen of etoposide + cisplatin, an immune checkpoint inhibitor (traplizumab) + FOLIFIRI (fluorouracil + calcium folinate + irinotecan) regimen was used.

**Outcomes:**

The patient achieved 20 months of clinical survival and ultimately died of myelosuppression.

**Lessons:**

The use of immune checkpoint inhibitors may become an effective tool in the treatment of neuroendocrine carcinoma of the gallbladder.

## 1. Introduction

Neuroendocrine tumors (NETs) are highly heterogeneous tumors that occur mainly in the gastrointestinal tract, lungs and pancreas, and other organs. They have a low incidence rate (approximately 0.74/100,000).^[1]^ Gallbladder neuroendocrine carcinoma (GB-NEC) are even rarer.^[2–4]^ GB-NECs are highly malignant, as liver metastasis and lymph node metastasis can occur early in the disease, resulting in a shorter survival time for patients compared to those with gallbladder adenocarcinoma. Unfortunately, as a rare disease, the diagnosis and treatment of this disease remain a challenge.^[5]^ We observed a patient with GB-NECs who was treated with immune checkpoint inhibitors combined with FOLIFIRI (fluorouracil + calcium folinate + irinotecan) and achieved good treatment outcomes. Although this is only a case report, it has profound implications.

## 2. Case report

**Figure 1.**
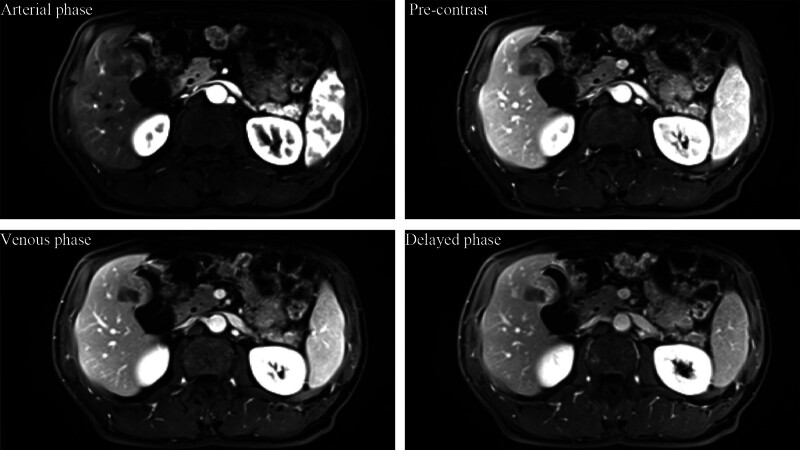
Magnetic resonance dynamic enhancement scan shows a gallbladder tumor in the body and base of the gallbladder, inhomogeneous enhancement in the venous phase, invasion of segment V of the liver, and fine line-like enhancement of the mucosal surface of the tumor.

**Figure 2.**
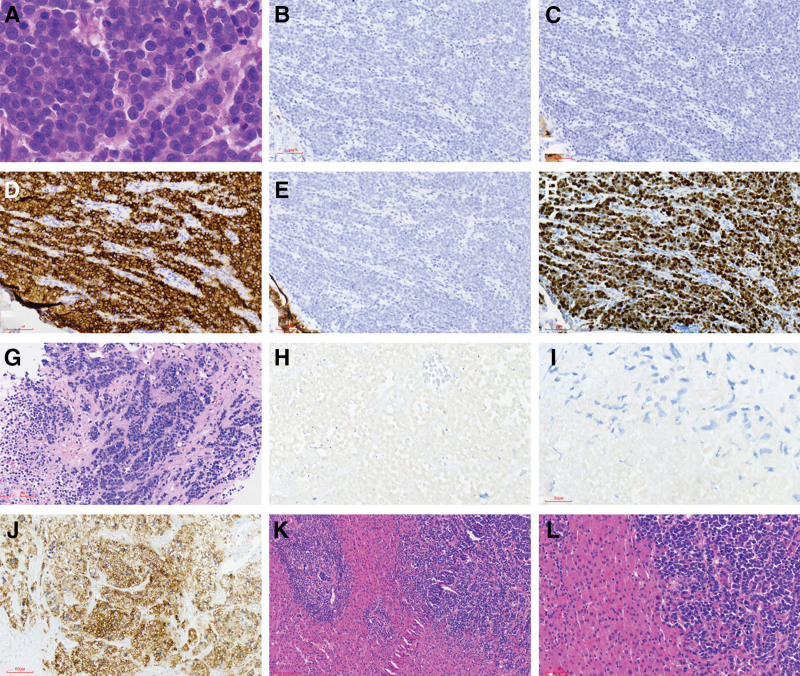
(A): Small cell neuroendocrine carcinoma of the gallbladder, with multiple mitotic figures visible in the field of vision, and active tumor growth (HE × 400); (B): Syn partially positive (immunohistochemistry × 200); (C): partial positive CgA (immunohistochemistry) × 200); (D): CD56 positive (immunohistochemistry × 200); (E): CK7 partially weakly positive (immunohistochemistry × 200); (F): Ki-67 90% positive (immunohistochemistry × 200); (G): small cell carcinoma, cell morphology consistent with primary small cell neuroendocrine carcinoma of the gallbladder (HE × 200); (H): Syn negative (immunohistochemistry × 200); (I): CgA negative (immunohistochemistry × 200); (J): CD56 positive (immunohistochemistry × 200); (K): The portal area of liver tissue around GB-NEC tumors can be infiltrated with a large number of lymphocytes (HE × 200); (L): GB-NEC tumor surrounding lymphocyte infiltration (HE) × 200).

The patient, a middle-aged male, was admitted for treatment due to right upper abdominal distension and discomfort for 10 days. On admission, physical examination revealed a flat abdomen with soft abdominal muscles. There was tenderness on deep palpation in the right upper abdomen, but no rebound tenderness. The liver and spleen were not palpable below the ribs, and no masses were found in the entire abdomen. Murphy sign was negative, and bowel sounds were normal. The patient had no significant medical history. After admission, an abdominal dynamic contrast enhanced magnetic resonance imaging (DCE-MRI) was performed, which revealed a soft tissue mass involving the gallbladder and liver, measuring approximately 4 × 4 cm with clear borders (Fig. 1). The mucosal surface of the gallbladder mass showed partial continuous linear enhancement. Soft tissue shadow was also observed in the porta hepatis area, suggesting invasion of the liver by a malignant gallbladder tumor with accompanying lymph node metastasis in the porta hepatis region. The patient’s laboratory tests upon admission revealed normal results for blood routine examination, liver function, and tumor markers. These included an alpha-fetoprotein (AFP) level of 1.91 ng/ml (reference range: 0–8.1 ng/ml), a carcinoembryonic antigen (CEA) level below 0.5 ng/ml (reference range: 0–10 ng/ml), carbohydrate antigen 199 (CA199) at 7.04 U/ml (reference range: 0–37 U/ml), carbohydrate antigen 125 (CA125) at 3.72 U/ml (reference range: 0–30.2 U/ml), carbohydrate antigen 72-4 (CA72-4) at 5.22 U/ml (reference range: 0–6.9 U/ml), and a neuron-specific enolase (NSE) level of 10.57 ng/ml (reference range: 0–15.2 ng/ml). The patient underwent radical resection of gallbladder cancer (liver segment IVb + V resection, gallbladder resection, and abdominal lymph node dissection) under general anesthesia with endotracheal intubation. The postoperative pathology report revealed small cell neuroendocrine carcinoma of the gallbladder, involving the full thickness of the gallbladder wall, extending into the liver tissue, infiltrating nerves, and displaying tumor emboli within blood vessels. The margins of the gallbladder and liver resections were clear. Pathological examination of lymph nodes showed metastasis to lymph nodes around the gallbladder (12C group 1/1, 8A group 1/2, and 12B group 1/2). Lymphocytic infiltration was observed in the perihepatic portal area. Immunohistochemistry results showed CD56(+), CgA(partial+), Syn(partial+), CK7(partial weak+), CK19(partial weak+), TTF(−), HepPar-1(−), glypican(−), AFP(−), and Ki-67 with a high proliferation index of 90% (Fig. 2A–F). The patient received 5 cycles of the EP regimen for chemotherapy. Follow-up computed tomography (CT) revealed liver metastases, multiple enlarged lymph nodes in the right supraclavicular region, and mediastinal lymph node enlargement. Subsequent tumor marker tests showed an AFP level of 1.91 ng/ml (reference range: 0–8.1 ng/ml), a CEA level below 0.5 ng/ml (reference range: 0–10 ng/ml), CA199 at 8.25 U/ml (reference range: 0–37 U/ml), CA72-4 at 13.24 U/ml (reference range: 0–6.9 U/ml), and a NSE level of 31.32 ng/ml (reference range: 0–15.2 ng/ml). The lymph node biopsy of the supraclavicular region revealed metastatic small cell neuroendocrine carcinoma. The immunohistochemistry results were as follows: CK(widespread+), CD56(+), CK8/18(+), CD117(partial weak+), CgA(−), Syn(−), TTF-1(−), PD-1(−), PD-L1:(tumor cells, 90%+; immune cells, 20%+), MLH1(+), MSH2(+), MSH6(+), PMS2(+), Ki-67: 80%. Based on the combination of imaging studies and biopsy results, disease progression (PD) was suspected (Fig. 2G–J). After receiving 3 cycles of FOLFIRI (lrinotecan + calcium folinate + fluorouracil) chemotherapy combined with Toripalimab treatment, the follow-up CT showed improved multiple lymph nodes enlargement in the right supraclavicular region compared to before, while the multiple intrahepatic metastases were not clearly identified, and there was enlargement of mediastinal lymph nodes. Taking into account the combination of imaging studies and biopsy results, partial response (PR) was considered.

The patient continued to receive FOLFIRI (Irinotecan + calcium folinate + fluorouracil) chemotherapy combined with Toripalimab treatment. The follow-up CT scan showed the disappearance of liver metastases and supraclavicular lymph node metastases, indicating sustained PR. The tumor markers also showed favorable results with carcinoembryonic antigen < 0.5ng/ml (0–10ng/ml), carbohydrate antigen 199 10.72U/ml (0–37U/ml), carbohydrate antigen 72-4 3.70U/ml (0–6.9U/ml), and NSE 9.45ng/ml (0–15.2ng/ml). After 6 months of treatment with FOLFIRI + Toripalimab, the patient underwent a follow-up 18F-fluoro-2-deoxy-2-D-glucose(18F-FDG PET/CT) scan, which revealed enlarged lymph nodes in the hepatogastric region, suggestive of metastasis. The patient underwent local radiotherapy + Toripalimab treatment, and a subsequent CT scan showed the disappearance of lymph nodes in the hepatogastric region. However, a follow-up CT scan indicated an increase in size and number of enlarged lymph nodes in the mediastinum, prompting a switch to chemotherapy with albumin-bound paclitaxel + irinotecan in combination with Toripalimab and sunitinib targeted therapy. Unfortunately, the patient succumbed to bone marrow suppression more than 20 months after the diagnosis of GB-NEC.

## 3. Discussion

The presence of neuroendocrine cells in the normal gallbladder is still a matter of controversy, and the origin of GB-NECs remains uncertain. However, studies have shown that 11.7% (12/102) of patients with gallbladder inflammation exhibit intestinal metaplasia in the gallbladder mucosa, and among these cases, the expression rates of neuroendocrine cell markers such as chromogranin A and serotonin are 83.3% and 50%, respectively.^[6]^ Based on these findings, it can be speculated that neuroendocrine cells in the gallbladder originate from chronic inflammation, with gallbladder inflammation being the most significant risk factor for GB-NECs.^[7]^ Approximately 22% of specimens from small cell-type GB-NECs show coexistence of adenocarcinoma components.^[8]^ Similarly, large cell-type GB-NECs often present with concurrent adenocarcinoma components.^[9]^ Neuroendocrine carcinomas of gastrointestinal origin exhibit characteristic molecular alterations similar to those seen in associated adenocarcinomas, whereas well-differentiated neuroendocrine tumors from the same origin do not exhibit these characteristic molecular changes.^[10,11]^ The expression rates of cytokeratin 7 (CK7) and cytokeratin 20 (CK20) in GB-NEC are similar to those in gallbladder adenocarcinoma.^[12]^ Based on this, some researchers believe that GB-NECs may originate from gallbladder adenocarcinoma. It has been confirmed that there is mutual transformation between adenocarcinoma components in gastrointestinal NETs and NETs components.^[13]^ There is also a viewpoint suggesting that neuroendocrine cells originate from multipotent stem cells within the gallbladder.^[14]^

According to the classification of digestive system tumors and NETs by the World Health Organization (WHO), NETs can be categorized based on their histological differentiation into well-differentiated NETs, poorly differentiated NETs, and mixed NETs. Well-differentiated NETs can further be classified into 3 grades, G1, G2, and G3, based on mitotic count and Ki-67 proliferation index.^[15]^ NETs derived from epithelial cells are classified into large cell-type NETs and small cell-type NETs, based on cell size. Neuroendocrine neoplasms (NETs) originating from the gallbladder are predominantly poorly differentiated NETs.

GB-NECs often lack specific clinical manifestations. A few functional GB-NECs may secrete histamine and serotonin, leading to symptoms resembling carcinoid syndrome, such as diarrhea, flushing, edema, and wheezing.^[16]^ The primary symptoms of GB-NECs commonly include right upper abdominal pain, often accompanied by abdominal distension and tenderness in the right upper quadrant. In this case, the patient presented with initial symptoms of right upper abdominal pain. These clinical manifestations cannot be distinguished from gallbladder adenocarcinoma or cholecystitis. Therefore, the diagnosis of GB-NECs heavily relies on pathological examination.

Most researchers believe that GB-NECs lack specific imaging features. Kim et al reported that compared to gallbladder adenocarcinoma, poorly differentiated GB-NECs liver metastases and lymph node metastases have a relatively larger volume and are more likely to show linear enhancement of the mucosal layer on enhanced CT (94.7% vs 10.6%, *P* < .0001).^[17]^ Bae et al reported that, in contrast to gallbladder adenocarcinoma, GB-NECs have the following characteristics on magnetic resonance imaging (MRI): clear tumor margins, intact tumor mucosal layers, and thickened enhanced tumor edges.^[18]^ Some researchers believe that GB-NECs originate from the deep inner layer of the mucosa and submucosa, while the mucosal layer can remain partially intact and show linear enhancement.^[18]^ 18F-FDG PET/CT can be used to assess local invasion and distant metastasis of GB-NECs, providing guidance for preoperative staging and treatment. The expression of somatostatin receptor subtype 2 and 5 (SSTR2/5) can be used to differentiate G3 NETs from NECs. Due to the lack of SSTR expression, neuroendocrine carcinomas cannot sufficiently accumulate the radiotracer during somatostatin analog-labeled PET/CT imaging.^[19]^ In this case, the patient’s upper abdominal enhanced MRI showed clear tumor margins and continuous linear enhancement of the mucosal surface (Fig. 1).

Due to the lack of specific clinical manifestations and tumor markers, the diagnosis of GB-NECs relies on pathological examination, especially immunohistochemical testing. Common immunohistochemical markers include CgA, Syn, CD56, CK, NSE, and Ki-67%. Among them, CgA and Syn have the highest positivity rates, reaching 91.9% and 84.8% respectively.^[20]^

There is currently no standard treatment for GB-NECs, and surgery remains the preferred treatment option. Surgical treatment options include laparoscopic cholecystectomy and radical cholecystectomy for gallbladder cancer. Laparoscopic cholecystectomy can achieve curative effects for patients with in situ carcinoma and GB-NEC stage I.^[16]^ However, due to the difficulty in diagnosing this type of tumor, most patients already have liver metastasis and adjacent lymph node metastasis at the time of diagnosis. Over 90% of small cell-type GB-NECs have distant or regional metastasis at the time of diagnosis.^[8]^ 66.2% of GB-NECs patients are diagnosed at stage IV, with lymph nodes, liver, and lungs being the most common sites of metastasis.^[21]^ For advanced stage GB-NECs patients, surgical resection combined with chemotherapy is superior to surgical resection alone. According to the study by Moskal et al, the median survival period for GB-NECs patients who only underwent gallbladder resection was 4 to 5 months, significantly shorter than the median survival period (13 months) for GB-NECs patients who underwent gallbladder resection combined with chemotherapy.^[8]^ High-grade NETs requires platinum-based chemotherapy regimens.^[22]^ There is no standardized chemotherapy regimen for GB-NECs patients, but treatment approaches combining therapies for small cell lung cancer and gastrointestinal or pancreatic NEC commonly use etoposide plus cisplatin as first-line treatment.^[23]^ However, researchers such as Iwasa and Morizane suggest that compared to NECs originating from the gastrointestinal or pancreatic tract, the etoposide plus cisplatin regimen demonstrates poorer antitumor efficacy in treating GB-NECs.^[24]^

In this case, the patient received postoperative chemotherapy with the EP regimen. After 5 cycles of the EP regimen, a follow-up CT scan showed liver metastasis, right supraclavicular lymph node metastasis, and mediastinal lymph node metastasis. Following local progression, the patient was started on the immune checkpoint inhibitor, Toripalimab in combination with the FOLFIRI regimen for chemotherapy. After 3 cycles of Toripalimab in combination with FOLFIRI, a follow-up CT scan indicated improved enlargement of multiple lymph nodes in the right supraclavicular region, while multiple liver metastases appeared less distinct compared to previous scans. The radiological findings suggested a PR in the patient’s condition. The patient continued with the current treatment regimen and achieved sustained relief for a period of 6 months. The patient’s overall survival extended beyond 20 months, significantly longer than the median survival period of 7 months for GB-NECs.^[4]^ The first immune checkpoint inhibitors approved by the US FDA for the treatment of NECs are nivolumab, used for refractory small cell lung cancer, and avelumab, used for metastatic Merkel cell carcinoma.^[25,26]^ There are limited reports on the use of immune checkpoint inhibitors in the treatment of GB-NECs. Only 1 case report by Jeena Chorath described a middle-aged female GB-NECs patient who achieved sustained relief of symptoms when treated with a combination of the PD-1 inhibitor(nivolumab), the CTLA-4 inhibitor(ipilimumab), and the EP chemotherapy regimen.^[27]^ Generally, immune therapy is believed to be most effective in the inflammatory tumor microenvironment. Therefore, patients tend to benefit more from immune checkpoint inhibitors when there is T lymphocyte infiltration in the tumor microenvironment and high expression of PD-L1 in tumor cells. In this case, the surgical resection specimen revealed abundant lymphocyte infiltration in the periportal liver tissue, and there was also lymphocyte infiltration around the tumor (Fig. 2K–L), indicating the presence of a greater number of antitumor immune cells in the tumor microenvironment. Additionally, PD-L1 expression was detected in the patient’s lymph node biopsy (tumor cells +, 90%).^[28]^ This may be the reason why the patient achieved a favorable treatment response when treated with Toripalimab.

## 4. Conclusion

The incidence of GB-NECs is low, and it lacks specific clinical manifestations. Its diagnosis relies on pathological examination. Surgical treatment is the preferred approach for GB-NECs, and combining surgical treatment with chemotherapy or other comprehensive therapies can achieve better treatment outcomes. Immune checkpoint inhibitors have also shown favorable treatment effects in GB-NECs and can be considered as a second-line treatment option for GB-NECs.

## Acknowledgments

Thanks to the Hepatobiliary Pancreatic Medical Center of Weifang People’s Hospital for providing us with patient information.

## Author contributions

**Conceptualization:** Hao Li, Chao Li, Liu Yanyan, Maohui Yin.

**Data curation:** Hao Li, Chao Li, Pan Lv, Liu Yanyan, Maohui Yin.

**Formal analysis:** Hao Li, Chao Li, Pan Lv, Liu Yanyan, Maohui Yin.

**Funding acquisition:** Maohui Yin.

**Investigation:** Hao Li, Chao Li, Pan Lv, Liu Yanyan, Maohui Yin.

**Methodology:** Hao Li.

**Project administration:** Hao Li, Liu Yanyan.

**Resources:** Hao Li, Liu Yanyan.

**Software:** Chao Li, Pan Lv, Liu Yanyan, Maohui Yin.

**Supervision:** Hao Li, Chao Li, Pan Lv, Liu Yanyan.

**Validation:** Hao Li.

**Writing – review & editing:** Hao Li.

**Writing – original draft:** Chao Li, Liu Yanyan, Maohui Yin.
